# Prognostic lncRNAs, miRNAs, and mRNAs Form a Competing Endogenous RNA Network in Colon Cancer

**DOI:** 10.3389/fonc.2019.00712

**Published:** 2019-08-06

**Authors:** Qian-Rong Huang, Xin-Bin Pan

**Affiliations:** ^1^Department of Neurosurgery, Affiliated Tumor Hospital of Guangxi Medical University, Nanning, China; ^2^Department of Radiation Oncology, Affiliated Tumor Hospital of Guangxi Medical University, Nanning, China

**Keywords:** colon cancer, lncRNA, miRNA, mRNA, TCGA, competing endogenous RNA/ceRNA

## Abstract

**Purpose:** To develop a multi-RNA-based model to provide survival risk prediction for colon cancer by constructing a competing endogenous RNAs (ceRNAs) network.

**Methods:** The prognostic information and expression of the lncRNAs, miRNAs, and mRNAs in colon cancer specimens from The Cancer Genome Atlas (TCGA) were assessed. Constructing prognostic models used the differentially expressed RNAs. Kyoto Encyclopedia of Genes and Genomes (KEGG) analyses and Gene Ontology were used to identify the functional role of the ceRNA network in the prognosis of colon cancer.

**Results:** Five lncRNAs (AC007384.1, AC002511.1, AC012640.1, C17orf82, and AP001619.1), 8 miRNAs (hsa-mir-141, hsa-mir-150, hsa-mir-375, hsa-mir-96, hsa-mir-107, hsa-mir-106a, hsa-mir-200a, and hsa-mir-1271), and 5 mRNAs (BDNF, KLF4, SESN2, SMOC1, and TRIB3) were highly correlated with tumor status and tumor stage. Three prognostic models based on the 5 lncRNAs, 8 miRNAs, and 5 mRNAs were constructed. The prognostic ability was 0.850 for the lncRNA-based model, 0.811 for the miRNA-based model, and 0.770 for the mRNA-based model. Patients with high-risk scores revealed worse overall survival. The KEGG pathways were significantly enriched in the “neuroactive ligand-receptor interaction.”

**Conclusion:** This study identified several potential prognostic biomarkers to construct a multi-RNA-based prognostic model for colon cancer.

## Introduction

Colon cancer is one of the most common malignancies worldwide. The morbidity and mortality of colon cancer are increasing rapidly (1). Radical resection combined with chemotherapy is used to improve survival (2). However, treatment outcomes remain unsatisfactory. The 5-years overall survival ranges from 50 to 65% (3–6). Moreover, treatment outcomes differ among patients in the same stage. Therefore, the identification of prognostic factors will lead to better interventions. Until now, the prognosis of colon cancer has mainly depended on the TNM stage. However, the TNM stage is based on anatomical information; it does not reflect the biological heterogeneity of colon cancer. Thus, understanding the molecular mechanism of the initiation and progression of colon cancer may provide effective prognostic biomarkers for patients with a poor prognosis.

Altered lncRNA expression is involved in the onset and development of colon cancer (7, 8). The mechanism of competing endogenous RNAs (ceRNAs) was proposed as a specific regulatory pathway of lncRNAs to explain how they exert their influence on protein levels (9, 10). Many studies reported that the ceRNA network might be a marker for prognosis in colorectal cancer (11–15). However, few studies have assessed the ceRNA network in colon cancer (16, 17). Li et al. (16) constructed a colon cancer associated ceRNA network which included 9 lncRNAs, 13 miRNAs, and 70 mRNAs. However, the study did not construct a prognostic model for colon cancer. The study assessed only the relationship between a single RNA and overall survival. As a multistep disease, colon carcinogenesis represents the accumulation of various genetic alternations and their complicated interactions. Thus, to assess the effect of a ceRNA network in colon cancer is important.

In this study, we comprehensively analyzed the prognostic roles of lncRNAs, miRNAs, and mRNAs in colon cancer to develop a multi-RNA-based model that can be used to predict survivals.

## Methods

### Data Processing

The clinical information and RNA data of colon cancer patients were downloaded from The Cancer Genome Atlas (TCGA) dataset (https://cancergenome.nih.gov/). MRNAs and lncRNAs with an expression value >10 were included. This study retained miRNAs with log2(RPM +1) >1 in more than 75% of samples. Inclusion criteria: (1) colon cancer patients with complete information on age, gender, tumor status, tumor stage, T stage, N stage, M stage, and histological type; (2) patients with complete lncRNA-seq, mRNA-seq, and miRNA-seq data; and (3) patients with a follow-up time of ≥30 days. Finally, 473 colon cancer tissues and 41 adjacent non-tumor tissue were examined.

### Differential Expressed RNAs Analysis

Using the edgeR package of R software to identify the differentially expressed lncRNAs, miRNAs, and mRNAs between colon cancer and adjacent normal tissues. Using fold change (FC) and associated *P-*values to assess expression differences. |FC| > 2 and *P* < 0.05 were considered statistically significant. The expression profiles of the lncRNAs, miRNAs, and mRNAs were converted to log2 (normalized value +1) values after normalization by edgeR.

### Survival Analysis

Patients with an overall survival time of ≥90 days were included in the survival analysis. Using univariate Cox regression to assess the associations between overall survival and the lncRNAs, miRNAs, and mRNAs. Genes with a *P* < 0.001 in the univariate Cox regression analysis were included into the multivariate Cox regression analysis. To constructed LncRNA, miRNA, and mRNA signature scores by multiplying the expression levels of independent biomarkers (*P* <0.01). Using a time-dependent receiver operating characteristic (ROC) curve analysis to assess the prognostic accuracy of each model. The area under the curve (AUC) was used to assess the prognostic accuracy. Using R software version 3.3.3 and the “survivalROC” package to perform the time-dependent ROC curve analysis. Finally, the associations between overall survival and the differentially expressed lncRNAs, miRNAs, and mRNAs were assessed in 347 colon cancer patients.

### Functional Analysis

Using Kyoto Encyclopedia of Genes and Genomes (KEGG) analyses and Gene Ontology (GO) to assess the functional role of the ceRNA network in the prognosis of colon cancer. Using the clusterProfiler package of R software to conduct these functional enrichment analyses. Using the GOplot package of R software to reveal the results of the KEGG and GO analyses. *P* < 0.05 was the cut-off value.

### CeRNA Network Construction

We constructed the ceRNA network based on the identified prognostic lncRNAs, miRNAs, and mRNAs. Using the miRcode (http://www.mircode.org/) database to predict lncRNA-miRNA interactions. Using TargetScan (http://www.targetscan.org/), miRTarBase (http://mirtarbase.mbc.nctu.edu.tw/php/index.php), and miRDB (http://www.mirdb.org/) to identify miRNA-mRNA interactions. We established a ceRNA regulatory network based on lncRNA-miRNA-mRNA axes by combining lncRNA-miRNA interactions with miRNA-target gene interactions. The ceRNA network is a complex posttranscriptional regulatory network. In the ceRNA network, lncRNAs, mRNAs, and other RNAs act as natural miRNA sponges to suppress miRNA functions by sharing one or more miRNA response elements. A lncRNA that harbors a similar sequence to its targeted miRNA functions as a ceRNA, regulates the level of the encoded protein and participates in the regulation of cell biology by sponging miRNAs. Using Cytoscape v3.6.0 to construct and visualize the co-expression network (18).

### Statistical Analysis

Using *t-*tests to assess the relationships between clinical characteristics and the gene expression profiles. Using the Kaplan-Meier method to assess survival rates. Using the log-rank test to assess differences between survival curves. R software (version 3.3.3) and GraphPad Prism 5 were used to plot the figures. Statistical analyses were conducted using SPSS 24.0 (SPSS, Inc., Chicago, IL, USA). A two-tailed *P* < 0.05 was considered statistically significant.

## Results

### Differentially Expressed RNAs

**Figure 1:**
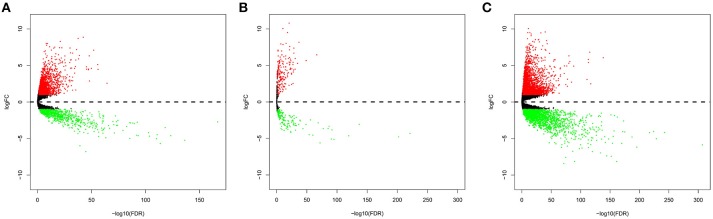
Volcano plot of the differentially expressed lncRNAs **(A)**, miRNAs **(B)**, and mRNAs **(C)**. The red points represent upregulated RNAs. The blue points represent downregulated RNAs.

In total, 2,146 upregulated and 820 downregulated lncRNAs (Figure 1A), 210 upregulated and 149 downregulated miRNAs (Figure 1B), and 3,031 upregulated and 2,339 downregulated mRNAs (Figure 1C) were identified.

### Survival-Associated RNAs

**Figure 2:**
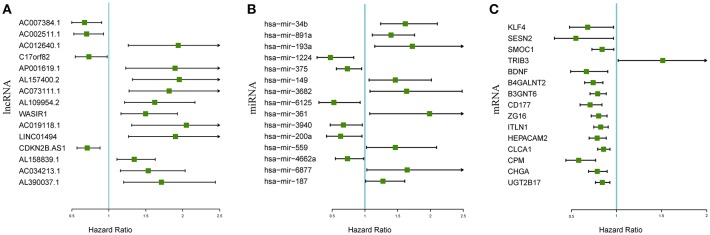
Forest plots of the hazard ratios of the top 15 survival-associated lncRNAs **(A)**, miRNAs **(B)**, and mRNAs **(C)**. A hazard ratio > 1 indicates the high-risk RNAs, and a hazard ratio < 1 indicates the protective RNAs.

**Figure 3:**
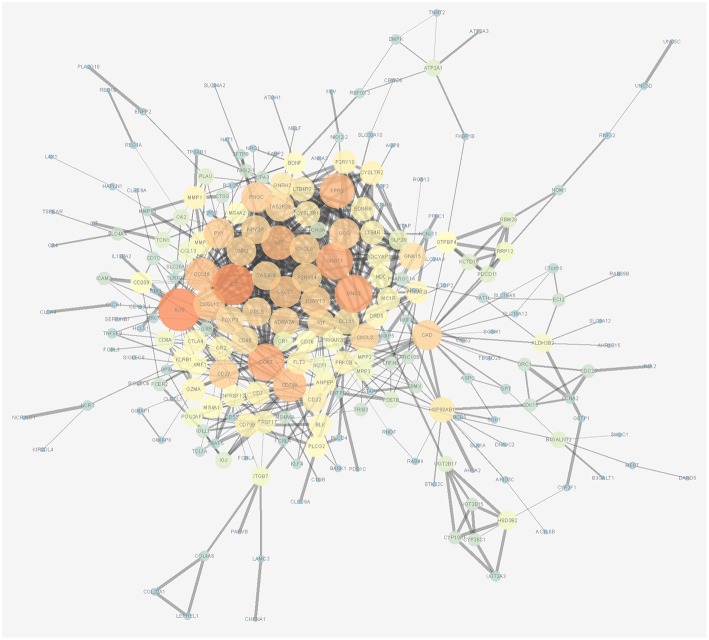
Protein-protein interaction network of overall survival-associated mRNAs generated by Cytoscape. The size and brightness of the circle represent the degree of the connection. The larger and brighter circles represent hub genes in the network. The thickness of the lines represents the combined score.

The associations between the differentially expressed lncRNAs, miRNAs, and mRNAs and overall survival were assessed in 347 colon cancer patients with at least 90 days of overall survival. This study performed a univariate survival analysis to identify overall survival related RNAs. The top 15 overall survival related lncRNAs, miRNAs, and mRNAs identified by the univariate analysis are presented in Figures 2A–C, respectively. Gene interaction networks were established with STRING using the overall survival-associated mRNAs (*P* < 0.001). The Cytoscape analysis revealed important cancer pathways and hub genes (Figure 3).

### Functional Analysis

**Figure 4:**
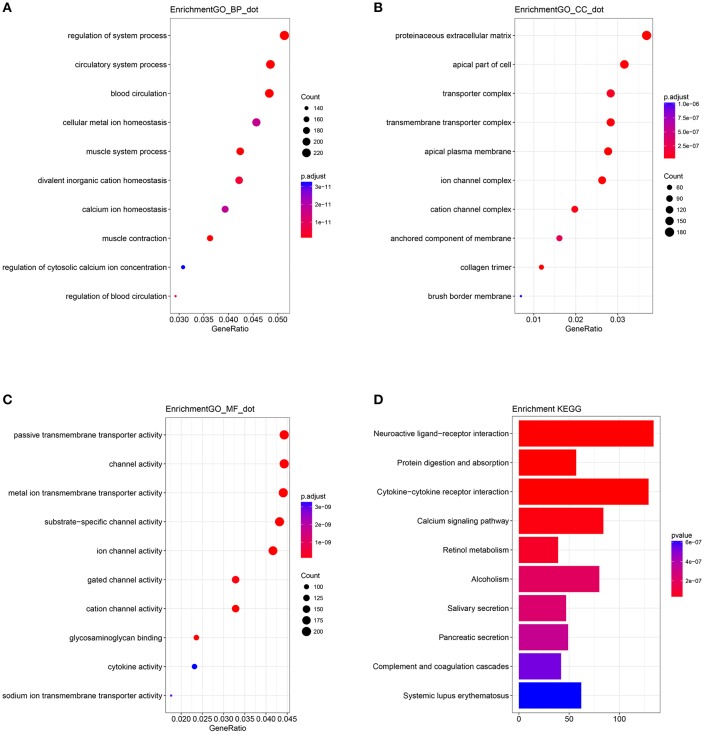
Gene Ontology (GO) terms of genes included in the competing endogenous RNA (ceRNA) network: **(A)** biological process, **(B)** cellular component, and **(C)** molecular function. **(D)** Kyoto Encyclopedia of Genes and Genomes (KEGG) pathways of genes included in the ceRNA network.

A biological enrichment analysis (through KEGG pathways and GO analysis) was performed to further analyse the biological functions of the ceRNAs. The results of GO analysis showed that these genes are involved in some regulation of system processes (Figure 4A).The cellular component process found that the target genes are mainly clustered into the proteinaceous extracellular matrix (Figure 4B). Regarding the molecular functions process, the target genes are significantly related to passive transmembrane transporter activity (Figure 4C). The KEGG pathways were mainly enriched in the “neuroactive ligand-receptor interaction” (Figure 4D).

### Predictive Model for Overall Survival

Using univariate and multivariate Cox regression analyses to evaluate the association between RNA expression and overall survival. RNAs with a *P* < 0.001 in the univariate Cox regression analysis were included into the multivariate Cox regression analysis. Five lncRNAs (AC007384.1, AC002511.1, AC012640.1, C17orf82, and AP001619.1), 8 miRNAs (hsa-mir-141, hsa-mir-150, hsa-mir-375, hsa-mir-96, hsa-mir-107, hsa-mir-106a, hsa-mir-200a, and hsa-mir-1271), and 5 mRNAs (BDNF, KLF4, SESN2, SMOC1, and TRIB3) were identified. Associations between the 5 lncRNAs, 8 miRNAs, and 5 mRNAs and clinical parameters were assessed. These genes showed a significant correlation with tumor status and tumor stage (Tables 1–3).

**Table 1:** Relationships between the expression of five lncRNAs and clinical parameters in the TCGA.

**Parameter**		**AC007384.1 (median)**	***P***	**AC002511.1 (median)**	***P***	**AC012640.1 (median)**	***P***	**C17orf82 (median)**	***P***	**AP001619.1 (median)**	***P***
Age	≤65	7.44 (3.67, 12.00)	0.490	3.57 (1.22, 8.62)	0.135	1.73 (0.58, 3.09)	0.859	6.49 (3.10, 12.04)	0.685	1.79 (0.68, 3.35)	0.977
	>65	6.59 (3.05, 11.98)		2.93 (0.59, 7.01)		1.72 (0.48, 2.99)		6.92 (3.17, 14.20)		1.94 (0.27, 3.45)	
Gender	Male	7.06 (3.31, 11.51)	0.632	2.86 (0.75, 6.70)	0.106	1.57 (0.52, 2.92)	0.526	6.63 (3.16, 13.44)	0.810	1.88 (0.35, 3.66)	0.524
	Female	7.24 (2.98, 12.70)		3.93 (1.17, 8.61)		1.83 (0.54, 3.21)		7.17 (3.09, 13.26)		1.80 (0.48, 3.33)	
Tumor status	With tumor	5.30 (2.33, 8.98)	0.014	2.80 (1.73, 6.70)	0.024	3.25 (1.22, 4.26)	0.003	5.02 (2.33, 9.14)	0.027	2.64 (1.35, 3.47)	0.017
	Tumor-free	7.95 (3.48, 13.09)		5.39 (0.84, 8.22)		1.69 (0.40, 2.85)		7.01 (3.18, 13.48)		1.37 (0.26, 3.29)	
Tumor stage	I–II	6.18 (3.41, 11.22)	0.035	3.45 (1.16, 8.03)	0.046	1.71 (0.44, 2.87)	0.028	8.97 (3.34, 14.39)	0.028	1.98 (0.35, 3.46)	0.037
	III–IV	9.21 (2.85, 12.87)		5.17 (0.73, 8.36)		2.74 (0.66, 3.48)		5.94 (2.67, 11.77)		1.04 (0.43, 3.41)	
T stage	T1–T2	7.10 (3.21, 11.49)	0.670	3.20 (1.24, 6.91)	0.688	1.66 (0.22, 2.87)	0.446	7.07 (3.51, 15.09)	0.254	1.99 (0.79, 3.43)	0.581
	T3–T4	7.18 (3.07, 12.07)		3.36 (0.80, 8.37)		1.75 (0.59, 3.20)		6.76 (3.06, 13.09)		1.79 (0.37, 3.44)	
N stage	N0	7.19 (3.26, 11.38)	0.891	3.45 (1.14, 8.36)	0.464	1.71 (0.45, 2.86)	0.256	6.88 (3.23, 14.17)	0.426	1.98 (0.41, 3.50)	0.381
	N1–N2	7.09 (2.85, 12.72)		3.08 (0.68, 8.02)		1.77 (0.62, 3.61)		6.02 (2.93, 12.84)		1.73 (0.42, 3.27)	
M stage	M0	7.26 (3.18, 11.74)	0.283	3.37 (0.66, 8.32)	0.980	1.73 (0.42, 2.87)	0.101	6.99 (3.20, 13.50)	0.246	1.66 (0.24, 3.31)	0.111
	M1	6.09 (2.57, 10.50)		2.99 (1.43, 7.77)		2.07 (1.04, 3.77)		5.16 (2.34, 9.10)		2.66 (1.60, 3.40)	
Histological type	Adenocarcinoma	6.39 (3.03, 11.38)	0.002	3.49 (0.95, 8.32)	0.581	1.77 (0.54, 3.20)	0.304	6.28 (2.93, 11.56)	<0.001	1.92 (0.34, 3.48)	0.913
	Mucinous adenocarcinoma	10.74 (4.91, 17.68)		2.66 (0.58, 8.47)		1.44 (0.58, 2.43)		13.95 (6.43, 20.09)		1.75 (0.88, 3.22)	

**Table 2:** Relationships between the expression of eight miRNAs and clinical parameters in the TCGA.

**Parameter**		**hsa-mir-141 (median)**	***P***	**hsa-mir-150 (median)**	***P***	**hsa-mir-96 (median)**	***P***	**hsa-mir-375 (median)**	***P***	**hsa-mir-107 (median)**	***P***
Age	≤65	4343.65 (2965.13, 5674.71)	<0.001	863.72 (454.01, 1618.58)	0.452	45.41 (28.40, 69.65)	0.225	47942.02 (26353.34, 100866.43)	0.534	181.32 (138.77, 228.51)	0.108
	>65	5049.49 (3765.06, 7106.80)		982.58 (537.87, 1548.57)		51.02 (32.03, 74.90)		55411.81 (29563.17, 94812.27)		172.03 (123.06, 212.21)	
Gender	Male	4798.04 (3601.21, 6412.57)	0.457	898.40 (452.72, 1638.84)	0.813	47.75 (29.40, 74.40)	0.588	54336.78 (28879.67, 94812.27)	0.664	186.42 (136.73, 225.93)	0.057
	Female	4554.13 (3430.89, 6301.60)		916.28 (531.20, 1516.84)		49.20 (32.74, 71.32)		52046.39 (28668.29, 105207.10)		169.08 (119.21, 213.01)	
Tumor status	With tumor	5316.60 (4050.86, 6184.96)	0.046	713.37 (373.21, 1115.95)	0.026	65.13 (35.50, 88.64)	0.017	39205.86 (22196.22, 81112.73)	0.038	157.63 (122.70, 237.68)	0.018
	Tumor-free	4554.13 (3430.89, 6119.76)		1074.85 (547.28, 1655.19)		45.43 (30.24, 68.48)		55730.34 (29114.78, 111363.28)		196.84 (131.50, 221.04)	
Tumor stage	I–II	5899.09 (3631.86, 7123.01)	0.008	1050.48 (517.60, 1591.66)	0.022	61.01 (29.90, 71.59)	0.046	76247.84 (28931.42, 111988.15)	0.034	151.16 (129.46, 212.67)	0.014
	III–IV	4361.46 (3280.84, 5674.71)		738.70 (456.42, 1516.84)		43.73 (33.64, 74.48)		47897.98 (27327.09, 89263.01)		193.81 (134.91, 223.91)	
T stage	T1–T2	4891.05 (3609.13, 6390.74)	0.444	1010.36 (632.14, 1484.62)	0.333	44.30 (27.14, 73.89)	0.399	50180.58 (23426.93, 107555.49)	0.475	164.18 (133.16, 204.31)	0.289
	T3–T4	4681.02 (3515.68, 6329.30)		869.48 (458.57, 1606.99)		50.38 (32.34, 73.99)		54587.03 (29646.94, 97855.21)		177.99 (130.50, 220.10)	
N stage	N0	4862.46 (3705.29, 7053.40)	0.007	961.55 (528.90, 1584.43)	0.220	47.48 (29.39, 71.29)	0.207	55455.48 (28513.05, 111272.45)	0.349	171.89 (129.94, 218.16)	0.364
	N1–N2	4343.65 (3289.96, 5634.19)		813.37 (441.13, 1521.86)		49.16 (34.50, 75.66)		47636.52 (29839.85, 83999.20)		183.81 (135.55, 221.49)	
M stage	M0	4665.83 (3498.43, 6327.50)	0.301	931.29 (516.40, 1633.02)	0.059	46.84 (30.66, 72.09)	0.405	54654.28 (27869.98, 98191.94)	0.189	171.16 (129.94, 211.40)	0.745
	M1	4373.90 (3329.64, 5260.47)		855.94 (385.03, 1140.76)		63.15 (29.79, 78.20)		38446.04 (30815.58, 73636.56)		182.77 (113.87, 228.76)	
Histological type	Adenocarcinoma	4715.26 (3560.05, 6337.34)	0.763	894.55 (506.45, 1603.69)	0.673	49.13 (30.52, 72.52)	0.861	49714.50 (27529.32, 87282.23)	0.012	173.69 (133.16, 220.54)	0.929
	Mucinous Adenocarcinoma	4575.52 (3228.10, 6359.86)		970.96 (399.45, 1435.92)		44.15 (31.99, 82.91)		105207.10 (46750.42, 163774.25)		176.84 (121.99, 209.17)	
**Parameter**		**hsa-mir-106a (median)**	***P***	**hsa-mir-200a (median)**	***P***	**hsa-mir-1271 (median)**	***P***				
Age	≤65	35.27 (21.54, 148.65)	0.004	6618.37 (4743.74, 9366.88)	0.680	4.52 (2.82, 8.25)	0.534				
	>65	59.15 (28.10, 212.59)		6831.14 (4948.76, 9542.22)		5.15 (2.52, 9.05)					
Gender	Male	40.42 (23.59, 210.86)	0.894	6377.21 (4928.21, 9383.77)	0.479	4.55 (2.64, 8.62)	0.696				
	Female	49.22 (24.74, 162.78)		6901.68 (4886.48, 9974.25)		4.97 (2.49, 8.85)					
Tumor status	With tumor	49.01 (29.10, 162.78)	0.821	6405.86 (4434.60, 8339.63)	0.223	5.17 (2.71, 7.41)	0.499				
	Tumor-free	44.15 (24.54, 179.91)		6746.91 (4886.48, 9523.17)		4.75 (2.58, 8.68)					
Tumor stage	I–II	48.95 (26.33, 201.04)	0.514	6862.89 (4998.25, 10049.39)	0.087	4.84 (2.57, 8.65)	0.765				
	II–IV	40.68 (24.59, 162.78)		6413.66 (4578.39, 8407.91)		4.66 (2.54, 8.68)					
T stage	T1–T2	59.52 (30.37, 228.71)	0.018	7311.11 (4750.56, 9600.96)	0.72	4.62 (2.92, 9.61)	0.392				
	T3–T4	40.60 (23.26, 149.75)		6605.67 (4948.08, 9422.44)		4.76 (2.54, 8.65)					
N stage	N0	48.95 (23.87, 194.55)	0.681	6844.90 (4961.17, 10134.23)	0.122	4.72 (2.59, 8.70)	0.914				
	N1–N2	40.24 (24.56, 166.60)		6542.43 (4672.52, 8381.86)		4.76 (2.60, 8.67)					
M stage	M0	49.20 (25.99, 208.86)	0.869	6605.67 (4965.03, 9281.02)	0.040	5.26 (2.96, 8.98)	0.87				
	M1	49.25 (25.69, 222.98)		4984.15 (3919.93, 9028.93)		5.18 (2.56, 9.26)					
Histological type	Adenocarcinoma	48.22 (26.38, 193.01)	0.041	6568.82 (4771.60, 9376.66)	0.064	4.75 (2.54, 8.62)	0.299				
	Mucinous Adenocarcinoma	30.86 (19.36, 143.93)		7621.25 (5407.56, 10935.37)		4.66 (3.19, 9.90)					

**Table 3:** Relationships between the expression of five mRNAs and clinical parameters in the TCGA.

**Parameter**		**BDNF (median)**	***P***	**KLF4 (median)**	***P***	**SESN2 (median)**	***P***	**SMOC1 (median)**	***P***	**TRIB3 (median)**	***P***
Age	≤65	21.94 (13.41, 32.10)	0.498	1926.9 (1137.48, 3194.51)	0.065	717.2 (549.84, 975.51)	0.704	65.41 (21.19, 460.94)	0.13	2257.02 (1466.81, 3570.39)	0.103
	>65	21.61 (12.25, 33.73)		2135.44 (1415.39, 3485.67)		773.23 (553.35, 925.67)		43.91 (15.15, 300.87)		2580.23 (1777.16, 3977.45)	
Gender	Male	21.89 (12.62, 32.18)	0.808	2072.26 (1243.52, 3579.47)	0.888	781.25 (546.56, 967.80)	0.719	45.35 (14.83, 328.70)	0.222	2481.51 (1719.51, 3965.87)	0.235
	Female	21.52 (13.31, 33.97)		2061.41 (1266.98, 3187.26)		751.7 (579.01, 921.05)		54.04 (19.62, 436.10)		2409.28 (1538.03, 3687.67)	
Tumor status	With tumor	25.2 (12.65, 35.89)	0.031	1470.83 (1056.38, 2119.47)	0.002	602.4 (463.86, 876.48)	0.029	36.51 (12.92, 313.23)	0.029	3173.08 (1814.36, 4067.39)	0.033
	Tumor-free	20.87 (13.30, 33.98)		2321.54 (1273.07, 3608.13)		883.79 (580.57, 969.70)		91.73 (20.55, 375.74)		2300.6 (1543.67, 3681.15)	
Tumor stage	I–II	21.43 (12.94, 30.70)	0.015	2130.86 (1328.92, 3595.92)	0.047	771.61 (580.29, 931.69)	0.021	47.68 (15.07, 328.59)	0.018	2408.85 (1559.30, 3591.86)	0.012
	III–IV	22.91 (13.07, 35.98)		1881.28 (1144.92, 2978.73)		720.08 (518.63, 924.44)		54.98 (19.00, 438.28)		3471.87 (1708.13, 4341.73)	
T stage	T1–T2	21.55 (12.92, 31.92)	0.781	2055.17 (1295.67, 3392.52)	0.549	708.46 (524.86, 907.00)	0.468	41.26 (19.77, 190.36)	0.489	2458.76 (1521.21, 3351.33)	0.608
	T3–T4	21.67 (13.00, 33.15)		2073.64 (1241.50, 3317.37)		764.83 (573.34, 958.38)		54.98 (15.50, 401.92)		2463.69 (1673.37, 3956.84)	
N stage	N0	21.32 (12.92, 30.06)	0.254	2147.92 (1358.99, 3610.62)	0.02	774.03 (583.19, 953.78)	0.19	47.68 (15.22, 316.05)	0.219	2408.85 (1607.91, 3647.15)	0.134
	N1–N2	23.06 (13.17, 36.17)		1825.69 (1154.93, 2902.08)		706.6 (505.17, 919.06)		62.79 (19.42, 453.54)		2671.87 (1707.82, 4374.76)	
M stage	M0	21.55 (12.49, 33.05)	0.428	2086.32 (1283.88, 3468.13)	0.024	760.55 (553.60, 929.69)	0.774	52.71 (19.37, 363.54)	0.881	2428.12 (1615.37, 3735.59)	0.015
	M1	22.51 (13.07, 41.18)		1540.46 (999.44, 2463.94)		749.79 (467.67, 978.45)		69.5 (22.08, 284.89)		3704.66 (1961.75, 5389.33)	
Histological type	Adenocarcinoma	21.6 (12.92, 32.17)	0.503	1983.13 (1220.34, 3176.10)	<0.001	772.43 (573.95, 961.12)	0.064	54.15 (15.94, 390.55)	0.258	2593.81 (1779.22, 4124.22)	<0.001
	Mucinous Adenocarcinoma	24.41 (13.16, 37.57)		3125.02 (1959.67, 5039.66)		658.91 (468.17, 867.89)		38.57 (20.66, 176.88)		1518.64 (1108.02, 2452.50)	

Predictive survival models were constructed based on the 5 lncRNAs, 8 miRNAs, and 5 mRNAs. The lncRNA-based model = [−0.365 × the expression value of AC007384.1] + [−0.403 × the expression value of AC002511.1] + [0.717 × the expression value of AC012640.1] + [−0.218 × the expression value of C17orf82] + [0.619 × the expression value of AP001619.1]. The miRNA-based model = [0.614 × the expression value of hsa-mir-141] + [−0.399 × the expression value of hsa-mir-150] + [0.431 × the expression value of hsa-mir-96] + [−0.329 × the expression value of hsa-mir-375] + [0.978 × the expression value of hsa-mir-107] + [−0.317 × the expression value of hsa-mir-106a] + [−0.668 × the expression value of hsa-mir-200a] + [0.613 × the expression value of hsa-mir-1271]. The mRNA-based model = [−0.4378 × the expression value of BDNF] + [−0.4078 × the expression value of KLF4] + [−0.5814 × the expression value of SESN2] + [−0.1876 × the expression value of SMOC1] + [0.3795 × expression value of TRIB3].

**Figure 5:**
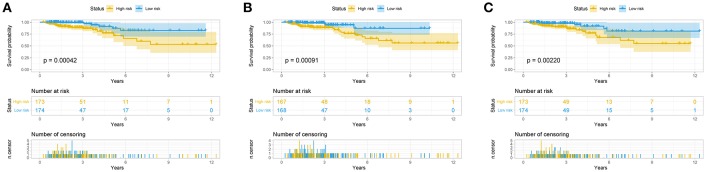
Predictive model for colorectal cancer patients. Overall survival curves according to lncRNA-based model **(A)**, the miRNA-based model **(B)**, and the mRNA-based model **(C)** of colon cancer patients with low or high risk.

**Figure 6:**
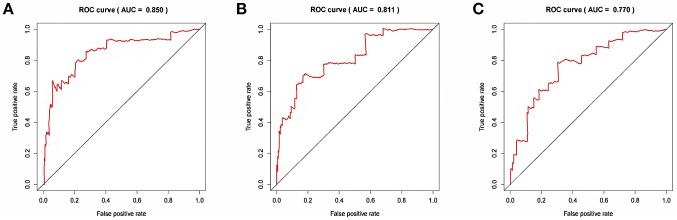
The receiver operating characteristic curve analysis of the risk score in predicting the overall survival curves of colon cancer patients: **(A)** the lncRNA-based model, **(B)** the miRNA-based model, and **(C)** the mRNA-based model.

The risk score for each patient was calculated. Then we ranked them by increasing scores. Based on the median risk score, patients were divided into high-risk and low-risk groups. Patients with high-risk scores had worse overall survival than those with low-risk scores in all three models (Figure 5). The AUC of the risk score revealed that all three models showed prognostic assessment ability: 0.850 for the lncRNA-based model, 0.811 for the miRNA-based model, and 0.770 for the mRNA-based model (Figure 6).

**Figure 7:**
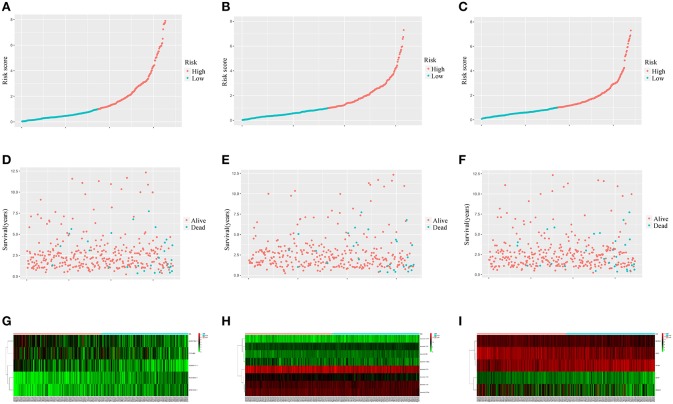
Performance of the prognostic models in distinguishing patients into low-risk and high-risk groups. The risk score distribution in the lncRNA-based model **(A)**, the miRNA-based model **(B)**, and the mRNA-based model **(C)**. The risk score distribution of survival status in the lncRNA-based model **(D)**, the miRNA-based model **(E)**, and the mRNA-based model **(F)**. Heatmap of the lncRNA **(G)**, miRNA **(H)**, and mRNA **(I)** expression profiles between the low risk score and high risk score groups.

Figure 7 showed that the scores assigned to each patient have a good prognostic assessment. Figure 7 also showed the expression patterns of these RNAs in the low-risk and high-risk groups.

### Survival-Related ceRNA Network

**Figure 8:**
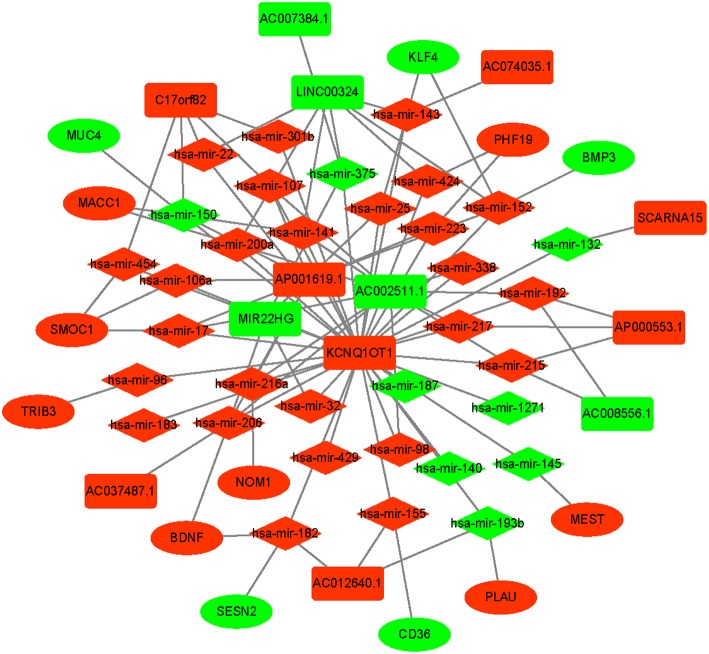
Prognostic competing endogenous RNA (ceRNA) network in colon cancer. The red indicates of the strongly expressed RNAs, and the green indicates the weakly expressed RNAs. The square represents lncRNAs, the diamond represents miRNAs, and the ellipse represents mRNAs.

**Figure 9:**
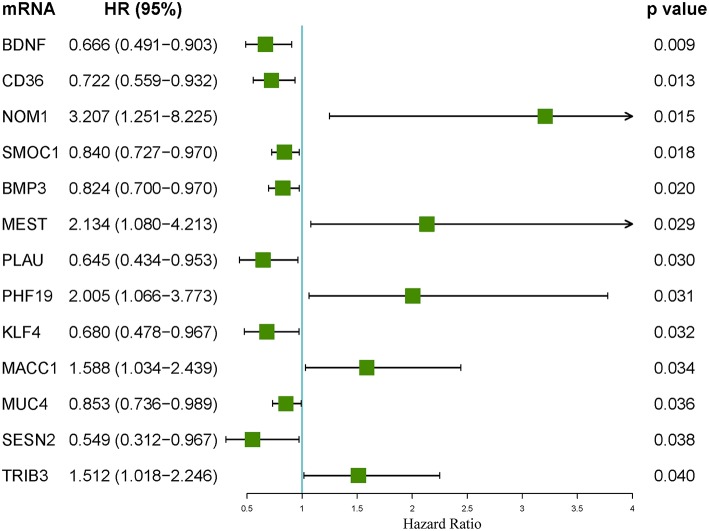
Forest plot of the hazard ratios of the mRNAs included in the competing endogenous RNA (ceRNA) network. A hazard ratio > 1 indicates the high-risk mRNAs, and a hazard ratio < 1 indicates the protective mRNAs.

An integrated lncRNA-miRNA-mRNA network was conducted by combining the lncRNA-miRNA interactions with the miRNA-mRNA interactions (Figure 8). The ceRNA network provides new insights into the diagnosis and prognosis of colon cancer. However, some molecules in this ceRNA network are not well understood in colon cancer. A comprehensive integrative analysis was performed. Figure 9 shows the mRNAs correlated with the prognosis of colon cancer patients.

## Discussion

This study identified distinct lncRNAs, mRNAs, and miRNAs to gain insights into the molecular events associated with colon cancer prognosis. Moreover, a prognostic ceRNA network was conducted to provide new dimensions of colon cancer prognosis.

Colon cancer is a heterogeneous disease with multiple molecular mutation. It is rarely ascribed to a single or a few genomic mutations alone (19). Until now, no single genetic “driver” was reported to be superior in evaluating aggressive disease (20). Hence, identifying effective prognostic markers is crucial for tailored treatment. Moreover, exploring the underlying regulatory network of biomarkers is essential for developing effective treatments.

Several studies have focused on a single type of lncRNA involved in colon cancer. Yue et al. (21) reported that FER1L4 expression was correlated with T stage, N stage, and vascular invasion. Moreover, significant differences in disease-free survival and overall survival were found in patients with both high miR-106a-5p expression and low FER1L4 expression compared with patients with low miR-106a-5p expression and high FER1L4 expression. Zhou et al. (22) reported that lincRNA-ROR expression correlated with vascular invasion and tumor stage. The knockdown of lincRNA-ROR restored the expression of miR-145. Moreover, knockdown of lincRNA-ROR had a significant impact on colon cancer cell invasion, proliferation, and migration. Patients with low miR-145 and high lincRNA-ROR had significantly poorer survivals than those with low high miR-145 and lincRNA-ROR. The depletion of miR-145 combined with the overexpression of lincRNA-ROR may play a crucial role on prognosis evaluation and treatment of colon cancer.

Other studies constructed a ceRNA network of colon cancer. Yan et al. (23) showed that lincRNA-ROR functions as a key ceRNA in colon cancer. Silencing lincRNA-ROR inhibited significantly colon cancer stem cell proliferation and increased the sensitivity to chemotherapy. Li et al. (16) constructed a colon cancer-associated ceRNA network. However, the study assessed only the relationship between a single RNA and overall survival and did not construct a prognostic model. Moreover, the study did not calculate the AUC of different prognostic RNAs. However, prognostic models based on multiple genes could provide more accurate predictions than single genes. Our study constructed a ceRNA network and indicated several new potential prognostic markers for colon cancer. Moreover, our study constructed three prognostic models based on the prognostic RNAs. The assessment ability was 0.850 for the lncRNA-based model, 0.811 for the miRNA-based model, and 0.770 for the mRNA-based model.

Previous studies have mainly focused on ceRNAs in colorectal cancer (11–15, 24, 25). Fan et al. (12) assessed the association between overall survival and a single RNA with different expression. However, colorectal carcinogenesis is the accumulation of various genetic alternations. Complicated interactions of genetic alteration play a key role. Thus, assessing the effect of a ceRNA network would provide better prognostic power. Zhang et al. (15) identified a 5-lncRNA (HOTAIR, H19, WT1-AS, LINC00488, and MIR31HG) model. The model was associated with the survival of colorectal cancer. The model showed a prognostic assessment ability of 0.675 for overall survival. In addition, Fan et al. (11) constructed a 6-lncRNA (RP11-798K3.2, RP11-785D18.3, RP1-170O19.17, RP11-167H9.4, RP11-481J13.1, and XXbac-B476C20.9) prognostic model. The AUC for the six-lncRNA model was 0.731. However, in our study, the prognostic power of lncRNA-based model was 0.850, which was higher than that of models described in previous studies. The higher prognostic power may be explained by two factors: (1) our study included patients only with colon cancer and not rectal cancer. (2) We only included patients with at least 90 days of overall survival in the survival analysis.

On the other hand, an increasing number of studies reported that colon cancer can be divided according to distal and proximal colon cancer (26–28). Our research did not detect a difference between colon cancer in the left and right colons. However, Qian et al. (17) found that 20 lncRNAs from the left colon and 25 lncRNAs from the right colon were associated with overall survival. In the ceRNA network, 18 lncRNAs, 22 miRNAs, and 57 mRNAs were included in left colon cancer, while 21 lncRNAs, 27 miRNAs, and 55 mRNAs were included in t right colon cancer. We further developed prognostic models to identify potential biomarkers for prognostic purposes.

In a ceRNA network, the implementers of molecular function are mRNAs. KEGG and GO analyses are used to identify pathways that are disturbed by the molecular cluster. The KEGG pathways in ceRNA network analysis revealed that the targeted genes are mainly enriched in the “neuroactive ligand-receptor interaction.” An imbalance in the homeostasis of the neuroactive ligand-receptor interaction may be the main influence of the ceRNA network on the prognosis of colon cancer. However, Li et al. (16) reported that the top six KEGG pathways are enriched significantly in cancer associated signaling pathways, such as “the Wnt signaling pathway, proteoglycans in cancer, and transcriptional misregulation in cancer.” The different pathways may be attributed to the genes involved in the different models and thus need to be further assessed.

This study had several limitations. First, several novel lncRNAs that were not reported previously need to be further explored to identify their potential molecular mechanisms. Second, the whole study was based on major online databases, and the prognostic models need to be verified in actual clinical operations to further confirm their effectiveness. Third, the information of colon cancer patients from the TCGA should be assessed with another experimental method. Fourth, it would be important to assess survival with therapy (chemotherapy or targeted therapy). However, the main purpose of this study was to develop a multi-RNA-based model to provide survival risk prediction for colon cancer by constructing a ceRNAs network. Then the model could be used to optimize treatments for patients with a high risk of poor survival. Moreover, the data of the associations between survival and therapy was unavailable in the database. Thus, we could not analysis survival with therapy. We are going to assess the RNAs of this study in clinic experiment. We hope the results of clinic experiment would provide a productive finding of the associations between survival and therapy.

In conclusion, this study constructed a ceRNA network that provides functional implications for the neuroactive ligand-receptor interaction of colon cancer. The results indicate several new potential prognostic markers for colon cancer.

## Data Availability

The raw data supporting the conclusions of this manuscript will be made available by the authors, without undue reservation, to any qualified researcher.

## Author Contributions

X-BP contributed to the conception of the study and performed the data analyses. Q-RH contributed to manuscript preparation and helped to perform the analysis with constructive discussions.

### Conflict of Interest Statement

The authors declare that the research was conducted in the absence of any commercial or financial relationships that could be construed as a potential conflict of interest.
